# Molecular epidemiology of clinical and carrier strains of methicillin resistant *Staphylococcus aureus *(MRSA) in the hospital settings of north India

**DOI:** 10.1186/1476-0711-5-22

**Published:** 2006-09-14

**Authors:** Javid A Dar, Manzoor A Thoker, Jamal A Khan, Asif Ali, Mohammed A Khan, Mohammed Rizwan, Khalid H Bhat, Mohammad J Dar, Niyaz Ahmed, Shamim Ahmad

**Affiliations:** 1 Microbiology Division, Institute of Ophthalmology, J. N. Medical College, Aligarh Muslim University, Aligarh, India; 2 Department of Microbiology, Sher-e-Kashmir Institute of Medical Sciences, Srinagar, India; 3 Division of Bacteriology, Department of Microbiology J. N. Medical College, Aligarh Muslim University, Aligarh, India; 4 Department of Biochemistry, J. N. Medical College, Aligarh Muslim University, Aligarh, India; 5 Laboratory of Molecular and Cell Biology, Centre for DNA Fingerprinting and Diagnostics Hyderabad, India

## Abstract

**Background:**

The study was conducted between 2000 and 2003 on 750 human subjects, yielding 850 strains of staphylococci from clinical specimens (575), nasal cultures of hospitalized patients (100) and eye & nasal sources of hospital workers (50 & 125 respectively) in order to determine their epidemiology, acquisition and dissemination of resistance genes.

**Methods:**

Organisms from clinical samples were isolated, cultured and identified as per the standard routine procedures. Susceptibility was measured by the agar diffusion method, as recommended by the Nat ional Committee for Clinical Laboratory Standards (NCCLS). The modified method of Birnboin and Takahashi was used for isolation of plasmids from staphylococci. Pulsed-field gel electrophoresis (PFGE) typing of clinical and carrier Methicillin resistant *Staphylococcus aureus *(MRSA) strains isolated during our study was performed as described previously.

**Results:**

It was shown that 35.1% of *Staphylococcus aureus *and 22.5% of coagulase-negative staphylococcal isolates were resistant to methicillin. Highest percentage of MRSA (35.5%) was found in pus specimens (n = 151). The multiple drug resistance of all MRSA (n = 180) and Methicillin resistant Coagulase-negative *Staphylococcus aureus *(MRCNS) (n = 76) isolates was detected. In case of both methicillin-resistant as well as methicillin-sensitive Saphylococcal isolates zero resistance was found to vancomycin where as highest resistance was found to penicillin G followed by ampicillin. It was shown that the major reservoir of methicillin resistant staphylococci in hospitals are colonized/infected inpatients and colonized hospital workers, with carriers at risk for developing endogenous infection or transmitting infection to health care workers and patients. The results were confirmed by molecular typing using PFGE by *Sma*I-digestion.

It was shown that the resistant markers G and T got transferred from clinical *S. aureus *(JS-105) to carrier *S. aureus *(JN-49) and the ciprofloxacin (Cf) and erythromycin (E) resistance seemed to be chromosomal mediated. In one of the experiments, plasmid pJMR1O from *Staphylococcus aureus *coding for ampicillin (A), gentamicin (G) and amikacin (Ak) resistance was transformed into *Escherichia coli*. The minimal inhibitory concentrations (MICs) for A and G were lower in *E. coli *than in *S. aureus*. However, the MIC for Ak was higher in *E. coli *transformants than in *S. aureus*.

**Conclusion:**

There is a progressive increase in MRSA prevalence and multi-drug resistance in staphylococci. Vancomycin is still the drug of choice for MRSA infections. The major reservoir of methicillin resistant staphylococci in hospitals is colonized/infected inpatients and colonized hospital workers. Resistance transfer from staphylococci to *E. coli *as well as from clinical to carrier staphylococci due to antibiotic stress seemed to be an alarming threat to antimicrobial chemotherapy.

## Background

Staphylococci are one of the important causes of human infections but are also found as non-pathogenic microorganisms in human samples [1-5]. The spectrum of *S. aureus *infections includes toxic shock syndrome [6], food poisoning, meningitis [6,7] as well as dermatological disorders ranging from minor infections and eczema to blisters and scalded skin syndrome [8]. Recent reports have begun to document infections caused by *Staphylococcus epidermidis*, such as bacterial endocarditis [9] prosthetic heart valve endocarditis [10], bacteraemia, surgical wound infections [11], intravascular catheters [12], post-operative endophathalmitis [13], conjunctivitis and keratitis [14]. Several other coagulase negative staphylococci (CNS) species have been implicated at low incidence in a variety of infections. The CNS species *Staphylococcus saprophyticus *was often regarded as a more important opportunistic pathogen than *S. epidermidis *in human urinary tract infections (UTIs), especially in young sexually active females. It was considered to be the second most common cause of acute cystatitis or pyelonephritis in these patients [15,16]. The major reservoir of Staphylococci in hospitals are colonized/infected in-patients and colonized hospital workers, with carriers at risk for developing endogenous infection or transmitting infection to health care workers and patients [3,17-23], while transient hand carriage of the organism on the hands of health care workers account for the major mechanism for patient to patient transmission [24].

Methicillin-resistant strains of staphylococci were identified immediately upon the introduction of methicillin into clinical practice. Methicillin-resistant *S. aureus *(MRSA) was initially identified for the first time in 1961 by Jevons [25,26]. Since then strains of methicillin-resistant *Staphylococcus aureus *and methicillin-resistant coagulase-negative staphylococci have spread worldwide [27,28] and have become established inside and outside of the hospital environment [29]. Already multiresistant to different classes of antibiotics, MRSA had been reported to acquire resistance to gentamicin and related aminoglycosides [30] therefore the treatment of infections due to these organisms and their eradication is very difficult. Constant monitoring of these strains is essential in order to control their spread in the hospital environment and transmission to the community.

The present study was undertaken with the aim of determining epidemiology of clinical and carrier staphylococci and molecular studies of their acquisition and dissemination of resistance in a hospital setting in northern India.

## Materials and methods

The study population (n = 750) was divided into healthy personnel (n = 175) and patients (n = 575) (including 50 medical personnel attending wound infections). For the healthy personnel, 125 hospital workers contributed nasal swabs and 50 hospital works contributed ocular swabs. None of the healthy personnel had taken any kind of antibiotics 7 days before the time of specimen collection. For the patients, 50 patients with wound infections admitted in Orthopedic Surgical Ward of J.N. Medical College, Aligarh Muslim University, Aligarh India, contributing nasal swabs besides pus culture and 525 subjects contributing different clinical sources were included. Informed consent was obtained from all the subjects before sample collection. The Ethical committees of Sher-e-Kashmir Institute of Medical Sciences, Srinagar and J.N. Medical College, Aligarh Muslim University, Aligarh India approved the study.

### Specimen collection

Organisms from clinical samples were cultured as per the routine procedures. The anterior nares were sampled as follows. A sterile cotton-tipped swab was moistened in a culture tube containing 2 ml of 0.1% buffered Tween 80. The swab was wrung out within the tube, swirled inside the anterior nares for five clockwise, and five counter clockwise rotations, reintroduced into the culture tube and wrung out. The ocular swabs were obtained as described previously [31]. Swabs were deposited in tubes containing 2 ml of detergent fluid (0.1% buffered Tween 80) serially diluted in 10 fold steps. 40 μl per dilution was then drop plated onto the 5% sheep blood agar plates and mannitol salt agar plates (Hi-Media Mumbai, India), and the plates were incubated for 24 h at 35°C and observed or the growth of suspected Staphylococci colonies. After the identity of the cultures was confirmed according to Bergey's manual including Gram's staining, catalase and coagulase test (slide and tube methods) [32,33], they were stored at -70°C in freezer vials containing 15% glycerol for further analysis.

### Antimicrobial susceptibility test

Susceptibility was measured by the agar diffusion method, as recommended by the National Committee for Clinical Laboratory Standards (NCCLS) [34], with the following discs; amikacin (Ak), 30 μg; ampicillin (A), 10 μg; cefazolin (Cz), 30 μg; cephalexin (Ce), 30 μg; cephotaxime (Cp), 30 μg; chloramphenicol (C), 30 μg; ciprofloxacin (Cf), 5 μg; clinadamycin (Cd), 2 μg; Co-trimoxazole (Co), 25 μg; fusidic acid (Fc), 10 μg; gentamicin (G), 10 μg; Imipenem (I), 10 μg; methicillin (M), 5 μg; penicillin G (P), 10 U; rifampin (R), 5 μg; roxithromycin (Ro), 30 μg; Tetracycline (T), 30 μg; and vancomycin, 30 μg; (Hi Media Mumbai, India). Methicillin-resistant Staphylococci were also tested for oxacillin resistance using the oxacillin-salt screening test performed according to NCCLS guidelines [35]. *S. aureus *ATCC^® ^29213, *S. aureus *ATCC^® ^43300; (American Type Culture Collection Manassas, VA USA) were used as the positive and negative controls respectively.

### Plasmid isolation, transformation and conjugation

The modified method of Birnboin and Takahashi was used for isolation of plasmids from staphylococci [36,37]. The resulting samples were separated on agarose gels and visualized under UV illumination after staining with ethidium bromide [38]. In order to study the transfer of resistance genes between different species of bacteria, transformation of plasmid DNA from *Staphylococcus aureus *(JMR-10) (A^R^, G^R^, Ak^R^) (Table 2) to *Escherichia coli *(DH5α) (A^S^, G^S^, Ak^S^) was performed as studied previously [39]. Plasmid DNA was isolated from JMR-10 strain and transformed to DH5α. Transformation was tested by antibiotic sensitivity tests as studied previously [34].

**Table 2:** Antibiotic resistance profiles of 61 strains of MRSA isolated in an orthopaedic surgical ward

**Pattern**	**Antibiotic resistance profiles of MRSA**	**No. of resistance markers**	**Strains**
1.	A,Cz,Ce,Cp,Co,G,I,M,P,R,Ro,T	12	JMR-1
2.	Ak,A,Cz,Ce,Cp,Cf,Cd,Co,G,I,M,P,Ro	13	JMR-2
3.	Ak,A,Cz,Ce,Cp,Co,G,I,M,P,Ro	11	JMR-3, 55, 33, 59
4.	A,Cz,Ce,Cp,Cd,Co,G,I,M,P,T	11	JMR-4
5.	A,Cz,Ce,Cp,Cd,Co,G,M,P	9	JMR-5
6.	A,Cz,Ce,Cp,Cf,Cd,Co,G,M,P,R,Ro	12	JMR-6, 38, 51
7.	A,Cz,Ce,Cp,Cf,Co,M,P,R,Ro	10	JMR-7
8.	Ce,Cd,Co,M,P,R	6	JMR-8
9.	A,Cz,Ce,Cp,C,Cd,Co,M,P,R	10	JMR-9
10.	Ak,A,Cz,Ce,Cp,Cf,Co,G,M,P,R,Ro,T	13	JMR-10
11.	Ak,A,Cz,Ce,Cp,Cd,Co,G,M,P,R,Ro	12	JMR-11, 53
12.	Ak,A,Cz,Ce,Cf,Cd,Co,G,I,M,P,R,Ro	13	JMR-12
13.	A,Cz,Ce,Cp,Cd,Co,G,M,P,R	10	JMR-13
14.	A,Cz,Ce,Cp,Co,M,P,R	8	JMR-14
15.	A,Cz,Ce,Cp,Cf,Co,G,M,P,R	10	JMR-15, 58
16.	A,Cz,Ce,Cp,Cf,Co,G,M,P	9	JMR-16
17.	Ak,A,Cz,Ce,Cp,Cd,Co,G,M,P,R	11	JMR-17
18.	A,Cz,Ce,Cp,Co,G,M,P,R	9	JMR-18
19.	Ak,A,Cz,Ce,Cp,Co,G,M,P,Ro	10	JMR-19, 61
20.	Ak,A,Cz,Ce,Cp,Cd,Co,G,I,M,P,R,Ro	13	JMR-20
21.	A,Ce,Cp,C,Co,G,M,P,T	9	JMR-21
22.	Ak,A,Cz,Cp,Co,G,I,M,P,Ro,T	11	JMR-22
23.	A,Cz,Ce,Cp,Co,G,M,P,Ro	9	JMR-23
24.	Ak,A,Cz,Ce,Cp,Cf,Cd,Co,G,M,P,Ro,T	13	JMR-24, 46, 48, 39
25.	Ak,A,Cz,Ce,Cp,C,Cf,Co,Fc,G,I,M,R,Ro,T	15	JMR-25
26.	A,Cz,Ce,Cp,Co,G,M,P,Ro,T	10	JMR-26, 52
27.	A,Cz,Ce,Cp,Co,G,M,P,Ro,T	10	JMR-27
28.	Ak,A,Cz,Ce,Cp,C,Cf,Cd,Co,Fc,G,I,M,P,R,Ro,T	17	JMR-28, 60
29	A,Cz,Ce,Cp,C,Cd,Co,G,M,P,R,Ro,T	13	JMR-29
30.	A,Cz,Ce,Cp,Co,G,I,M,P,R,Ro	11	JMR-30
31.	Ce,Cp,Cd,Co,M,P	6	JMR-31, 56
32.	A,Cz,Ce,Cp,Cf,Co,G,M,P,R,Ro,T	12	JMR-32
33.	A,Cz,Ce,Cp,C,Cf,Cd,G,Co,I,M,P,Ro,T	14	JMR-34
34.	A,Cz,Ce,Cp,C,Co,G,M,P,Ro,T	11	JMR-35
35.	Ak,A,Cz,Ce,Cp,C,Co,G,M,P,R,Ro	12	JMR-36, 49
36.	Cz,Ce,Cp,Cd,Co,M,P	7	JMR-37, 45, 50, 54
37.	A,Cz,Ce,Cp,Cf,Co,M,P,R	9	JMR-40
38.	A,Cz,Ce,Cp,C,Cf,Cd,Co,Fc,G,I,M,P,R,Ro,T	16	JMR-41
39.	Ak,A,Cz,Cp,I,M,P,Ro	8	JMR-42
40.	A,Cz,Ce,Cp,Cd,Co,G,M,P,R,Ro	11	JMR-43
41.	A,Cz,Ce,Cp,Cf,Co,G,M,P,Ro	10	JMR-44
42.	A,Cz,Ce,Cp,Co,G,M,P,Ro	9	JMr-47, 57

For resistance transfer to be studied through cell – to – cell contacts between clinical and carrier *S. aureus *strains, the conjugation was performed through mixed culture tests as studied previously [33,39]. Here *S. aureus *strains were isolated from a patient having postoperative ocular infection admitted in the postoperative ward of the Institute of Ophthalmology. JN-49 strain (A^R^, P^R^, Cf^R^, E^R^, G^S^, T^S^) was isolated from the nose of the same patient having postoperative ocular infection and JS-105 (T^R^, E^R^, G^R^, A^S^, P^S^, Cf^S^) was isolated from ocular swab.

### PFGE

Chromosomal DNA from MRSA isolates was prepared in agarose blocks and was cleaved with *Sma*I (Bangalore Genei Pvt. Ltd. India) as described by Bannerman et al [40]. PFGE typing of clinical and carrier MRSA strains isolated during our study was performed as described previously [39,41]. For restriction endonuclease digestion, approximately 1 to 1.5 mm of a plug was cut and incubated with 250 μl of restriction buffer containing 20 U of *Sma*I at 25°C for 4 h. After DNA digestion, the agarose plugs were incubated with 1 ml of TE buffer at 37°C for 1 h. The plugs were then inserted into 1% agorose gel in 0.5× TBE buffer, and restriction fragments were separated using a contour-clamped homogeneous electric field system (CHEF-DRII; Bio-Rad, Laboratories). Electrophoresis was performed using the following conditions: Block 1: initial switch time 5 sec; final switch time 15 sec; run time 10 h; voltage 200 V or 6 V/cm. Block 2: initial switch time 15 sec, final switch time 60 sec. run time 13 h, voltage 20 V or 6 V/cm.

## Results

### Age and gender distribution

Of the 750 subjects studied the male: female ratio was 52.5:47.5, of whom 175 were normal hospital workers (Table 5). The age range was 5 to 75 years. Of these 12 cases of mastatitis belonged exclusively to females and 18 cases of prostatitis to men. Of patients having wound infections (n = 122), the highest prevalence of Staphylococci (27%) was found in the age group of 45–64 years and so was the case with patients having urinary tract infections (32.6%). In this study Staphylococcal conjunctivitis was found equally prominent in the age group of 5–14 (23.1%) and 45–64 (23.1%) years. Of the 175 normal hospital workers, the highest number of carrier Staphylococci (48%) were isolated from the subjects having age between 25 and 44 years where as the age group 5–14 years contributed none. In the present study we were having no information regarding the age of 52 subjects and so they were categorized as unknown age group.

**Table 5:** Age and gender distribution of staphylococcal infected/colonized subjects (n = 750) according to clinical diagnosis

Clinical Diagnosis n = 750 (100%)	Age (Y)											
	
	5–14 n = 63 (%)		15–24 n = 133 (%)		25–44 n = 224 (%)		45–64 n = 182 (%)		>65 n = 96 (%)		Unknown^a ^n = 52 (%)	
	
	Male n = 31(49.2)	Female n = 32 (50.8)	Male n = 67(50.4)	Female n = 66(49.6)	Male n = 116(51.8)	Female n = 108(48.2)	Male n = 101(55.5)	Female n = 81(44.5)	Male n = 56(58.3)	Female n = 40(41.7)	Male n = 23(44.2)	Female n = 29(55.8)
Wound infection n = 122 (16.2)	8 (12.7)	5 (7.9)	16 (12.0)	14 (10.5)	16 (7.1)	7 (3.1)	17 (9.3)	16 (8.8)	9 (9.4)	7 (7.3)	2 (3.8)	5 (9.6)
Postoperative infection n = 35 (4.7)	1 (1.6)	2 (3.2)	2 (1.5)	1 (0.7)	2 (0.9)	8 (3.6)	4 (2.2)	5 (2.7)	8 (8.3)	2 (2.1)	-	-
Bacteraemia n = 25 (3.3)	1 (1.6)	1 (1.6)	-	2 (1.5)	4 (1.8)	2 (0.9)	7 (3.8)	4 (2.2)	1 (1.0)	-	2 (3.8)	1 (1.9)
Pneumonia n = 21 (2.8)	5(7.9)	1 (1.6)	1 (0.7)	-	1 (0.4)	1 (0.4)	1 (0.5)	2 (1.1)	3 (3.1)	1 (1.0)	3 (5.8)	2 (3.8)
Septicaemia n = 15 (2.0)	-	-	1 (0.7)	1 (0.7)	1 (0.4)	2 (0.9)	2 (1.1)	2 (1.1)	1 (1.0)	-	2 (3.8)	3 (5.8)
Urinary tract infections n = 89 (11.9)	-	1 (1.6)	4 (3.0)	5 (3.8)	10 (4.5)	15 (6.7)	14 (7.7)	15 (8.2)	9 (9.4)	11(11.5)	-	5 (9.6)
Mastatitis n = 12 (1.6)	-	-	-	2 (1.5)	-	8 (3.6)	-	2 (1.1)	-	-	-	-
Prostatitis n = 18 (2.4)	-	-	1 (0.7)	-	5 (2.2)	-	9 (4.9)	-	3 (3.1)	-	-	-
Conjunctivitis n = 52 (6.9)	7 (11.1)	5 (7.9)	5 (3.8)	4 (3.0)	1 (0.4)	4 (1.8)	7 (3.8)	5 (2.7)	4 (4.2)	5 (5.2)	3 (5.8)	2 (3.8)
Corneal ulcer n = 20 (2.7)	1 (1.6)	-	2 (1.5)	-	2 (0.9)	1 (0.4)	2 (1.1)	3 (1.6)	3 (3.1)	2 (2.1)	1 (1.9)	3 (5.8)
Endophthalmitis n = 11 (1.5)	-	-	1 (0.7)	-	-	1 (0.4)	2 (1.1)	2 (1.1)	2 (2.1)	1 (1.0)	1 (1.9)	1 (1.9)
Other^b ^n = 110 (14.7)	8 (12.7)	13 (20.6)	11 (8.3)	13 (9.8)	12 (5.3)	8 (3.6)	12 (6.6)	8 (4.4)	11 (11.5)	9 (9.4)	3 (5.8)	2 (3.8)
Normal^c ^n = 175 (23.3)	-	-	20 (15.0)	18 (13.5)	56 (25.0)	46 (20.5)	16 (8.8)	15 (8.2)	-	-	2 (3.8)	2 (3.8)
Unknown^d ^n = 45 (6.0)	-	4 (6.3)	3 (2.2)	6 (4.5)	6 (2.7)	5 (2.2)	8 (4.4)	2 (1.1)	2 (2.1)	2 (2.1)	4 (7.7)	3 (5.8)

^a^Age not recorded for 52 subjects.

^b^Abscess or osteomyelitis, cystitis, peritonitis, endocarditis, toxic shock syndrome, keratitis, etc.

^c^Strains isolated from nose of hospital workers attending wound infections (n=50), nose of hospital workers (n=75) and normal eyes of hospital  workers (n=50) respectively in Orthopaedic Surgical Ward of J.N Medical College, A.M.U., Aligarh, S.K. Institute of Medical Sciences Kashmir  and Surgical Wards of Institute of Ophthalmology, A.M.U. Aligarh.

^d^Clinical diagnosis not recorded for 45 subjects.

### Frequency of staphylococcal isolates among clinical sources

**Figure 1:**
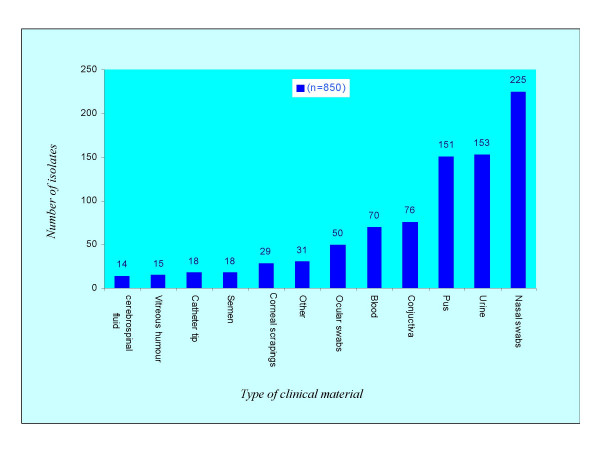
**Distribution of staphylococcal isolates among the clinical sources**. Out of 151 isolates from pus, 115 were *S. aureus *and 36 were coagulase-negative staphylococci (CNS). Likewise, of 153 isolates from urine, 77 were CNS and 76 were *S. aureus*. Of the 225 nasal swabs of hospital workers and patients studied, 175 (34.1%) were *S. aureus *and 50 (14.8%) were CNS.

Of the 850 clinical strains of staphylococci studied (Table 1), 60.3% were *Staphylococcus aureus *and 39.7% coagulase-negative staphylococci. Of these 35.1% were methicillin-resistant *Staphylococcus aureus *(MRSA) and 22.5% were methicillin-resistant coagulase-negative staphylococci (MRCNS). The highest number of *S. aureus *strains was isolated from pus (22.7%) followed by urine (14.8%) and blood (11.3%) (Figure 1). Likewise, the highest number of coagulase-negative staphylococcal isolates was found in urine (22.8%) followed by conjunctivitis (16.3%) and pus (10.7%). Of the 225 nasal swabs of hospital workers and patients studied, 34.1% were *S. aureus *and 14.8% were coagulase-negative staphylococci, where as from ocular swabs (n = 50) of hospital workers all the strains studied were coagulase-negative staphylococci. Of the 180 MRSA strains the highest number was found in pus (35.5%) followed by urine (16.1%) and blood (9.4%). In case of nasal swabs of hospital workers and patients (n = 225) the frequency of MRSA isolates was 28.9% that is equivalent to MRSA carrier rate of 23.1%. Of the 76 MRCNS studied the highest number was obtained from urine (25.0%) followed by conjunctiva (18.4%) and nasal swabs of patients having ocular infections (14.5%), where as 10.5% of MRCNS were isolated from ocular swabs of hospital workers.

**Table 1:** Resistance profiles^a^ of staphylococcal isolates (n = 850) from eyes and other clinical sources in health and disease

Antimicrobial Agent (n = 18)^b^	Percentage of Resistance^c ^Displayed by					
	
	*S. aureus *n = 513 (%)	Clinical *S. arueus *n = 338(%)	Carrier *S. arueus *n = 175 (%)	Coagulase-negative Staphylococci (CNS) n = 337 (%)	Clinical CNS n = 237 (%)	Carrier CNS n = 100 (%)
Amikacin	65 (12.7)	47 (13.9)	18 (10.3)	27 (8.0)	27 (11.4)	0 (0.0)
Ampicillin	413 (80.5)	275 (81.4)	138 (78.8)	241 (71.5)	183 (77.2)	58 (58.0)
Cefazolin	190 (37.0)	131 (38.8)	59 (33.7)	80 (23.7)	64 (27.0)	16 (16.0)
Cephalexin	237 (46.2)	165 (48.8)	72 (41.1)	86 (25.5)	68 (28.7)	18 (18.0)
Cephotaxime	207 (40.3)	145 (42.9)	62 (35.4)	78 (23.2)	68 (28.7)	16 (16.0)
Chloramphenicol	22 (4.3)	16 (4.7)	6 (3.4)	17 (5.0)	12 (5.1)	5 (5.0)
Ciprofloxacin	103 (20.1)	81 (24.0)	22 (12.6)	75 (22.2)	56 (23.6)	19 (19.0)
Clindamycin	79 (15.4)	60 (17.7)	19 (10.9)	71 (21.1)	56 (23.6)	15 (15.0)
Co-trimoxazole^d^	256 (50.0)	175 (51.8)	81 (46.3)	136 (40.3)	104 (43.9)	32 (32.0)
Fusidic acid	18 (3.5)	14 (4.1)	4 (2.3)	12 (3.6)	8 (3.4)	4 (4.0)
Gentamicin	172 (33.5)	122 (36.1)	50 (28.6)	90 (26.7)	72 (30.4)	18 (18.0)
Imipenem	136 (26.5)	97 (28.7)	39 (23.3)	68 (20.2)	54 (22.8)	14 (14.0)
Methicillin^e^	180 (35.1)	128 (37.9)	52 (29.7)	76 (22.5)	63 (26.6)	13 (13.0)
Penicillin G	472 (92.0)	313 (92.6)	159 (90.9)	303 (89.9)	222 (93.7)	81 (81.0)
Rifampin	97 (18.9)	68 (20.1)	29 (16.6)	41 (12.2)	33 (13.9)	8 (8.0)
Roxithromycin	146 (24.6)	103 (30.5)	43 (24.6)	53 (15.7)	48 (20.2)	8 (8.0)
Tetracycline	61 (11.9)	43 (12.7)	18 (10.3)	24 (7.1)	21 (8.9)	3 (3.0)
Vancomycin	0 (0.0)	0 (0.0)	0 (0.0)	0 (0.0)	0 (0.0)	0 (0.0)

^a^In accordance to Performance Standards for Antimicrobial Disk Susceptibility Tests, NCCLS (1998, 1999, 2000, 2002).

^b^Atleast one antimicrobial agent assayed from a particular class of antibiotics.

^c^The intermediate resistant category was included in sensitive category.

^d^(trimethoprim/sulphamethoxazole).

^e^Methicillin resistance was confirmed by Oxacillin Salt-agar screening – plate procedure

### Resistance Profiles of Staphylococcal Isolates

The resistance patterns of staphylococcal isolates (n = 850) to 18 antimicrobial agents are shown in Table 1. Vancomycin appeared to be the most effective drug. In case of *Staphylococcus aureus *(n = 513), the highest resistance was observed to penicillin G (92.0%) followed by ampicillin (80.5%) and co-trimoxazole (50.0%). Though the highest resistance in coagulase-negative staphylococci (n = 337) was shown to penicillin G (89.9%) but it was lesser by (2.1%) as compared to its resistance in *Staphylococcus aureus*, followed by the same pattern as to ampicillin (71.5%) and co-trimoxazole (40.3%).

As far as the clinical versus carrier staphylococcal islates are concerned the general observation was that the resistance in carrier isolates was lesser than clinical strains in case of all antibiotics. In clinical (n = 338) and carrier (n = 175) isolates of *S. aureus *the highest resistance was found to penicillin G (92.6% versus 90.60%) followed by ampicillin (81.4% versus 78.8%) and co-trimoxazole (51.8% versus 46.3%) respectively. The least resistance was shown to fusidic acid (4.1% versus 2.3) followed by chloramphenicol (4.7% versus 3.4%), tetracycline (12.7% versus 7.1%) and amikacin (13.9% versus 10.3%) respectively in clinical and carrier isolates of *S. aureus*. Almost similar trend was observed in clinical (n = 237) and carrier (n = 100) isolates of coagulase-negative staphylococci, with the exception that in carrier isolates zero resistance to amikacin was seen.

### Comparative multi-drug resistance patterns of methicillin-resistant and methicillin-sensitive clinical and carrier isolates

**Figure 2:**
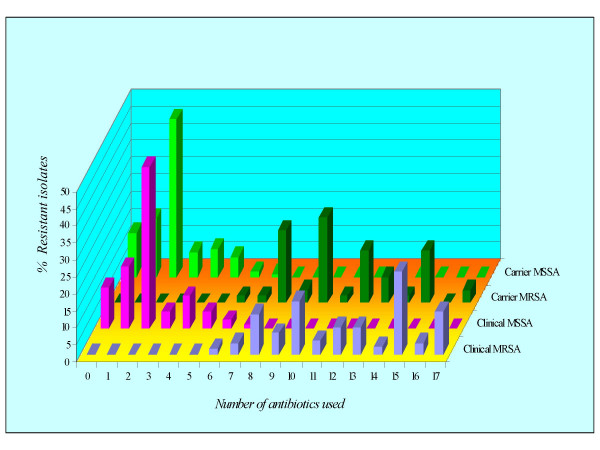
Occurrence of multidrug resistance in methicillin-resistant and methicillin-sensitive *S. aureus *clinical and carrier isolates.

Resistance to 4 antibiotics or more was observed in both *S. aureus *and Coagulase-negative staphylococci. In case of MRSA isolates, no strain showed resistance to 5 or less than 5 antibiotics, where as all MRCNS strains showed resistance to 8 or more than 8 antibiotics. As far as the clinical and carrier MRSA are concerned, in the present study, both types of strains showed resistance to 6 or more than 6 antibiotics assayed (Fig 2). In case of clinical and carrier methicillin-sensitive *S. aureus (*MSSA) isolates, no strain showed resistance to more than 7 antibiotics.

**Figure 3:**
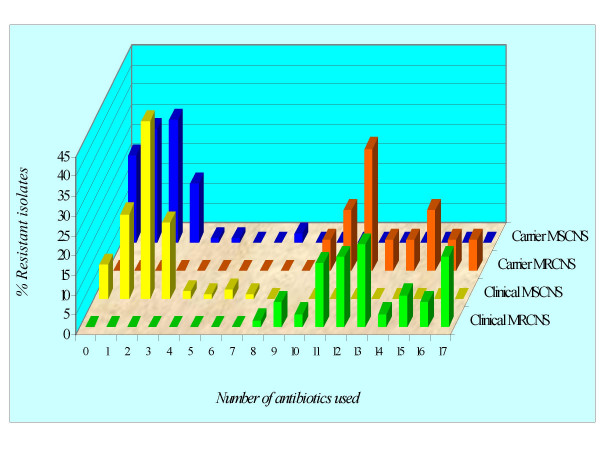
Occurrence of multidrug resistance in methicillin-resistant and methicillin-sensitive coagulase-negative staphylococcal clinical and carrier Isolates.

Almost same trend of multidrug resistance patterns was observed in case of clinical and carrier, MRCNS and MSCNS isolates from eye and other clinical sources in health and diseases (Fig 3).

### Antibiotic resistance profiles of MRSA isolates in an orthopedic surgical ward

In this study, 61 isolates turned to be MRSA, were isolated in an Orthopaedic Surgical Ward from wound infections (n = 56); their nose (n = 50); and nose of hospital workers attending wound infections (n = 50). As depicted in Table 2, MRSA isolates, JMR-8, 31, 56 showed resistance to 6 antibiotics; and one strain showed resistance to 16 antibiotics (JMR-41), and two strains showed resistance to maximum of 17 antibiotics (JMR-28, 60) assayed in the present study. Antibiotic resistance patterns, 3, 24, and 36 were the most common profiles found by 4 strains each in this study.

**Table 3:** PFGE patterns of 61 MRSA isolates

**Banding pattern**	**Strains**	**Number of isolates**
A	JMR-1, 2, 3, 4, 6, 38, 55	7
B	JMR-5, 14, 18	3
C	JMR-9, 13, 19, 26, 52	5
D	JMR-16, 25, 29	3
E	JMR-24, 46, 48, 39	4
F	JMR-31, 56	2
G	JMR-37, 50, 54, 15, 58	5
H	JMR-21, 40, 42, 44, 59, 61	6
I	JMR-8	1
J	JMR-17, 20, 23, 27, 30, 32,	6
K	JMR-11, 12, 28, 41, 53 60,	6
L	JMR-22, 33, 34, 35, 36	5
M	JMR-43, 45, 51	3
N	JMR-7, 10, 47, 49, 57	5

### PFGE profiles of MRSA

**Figure 4:**
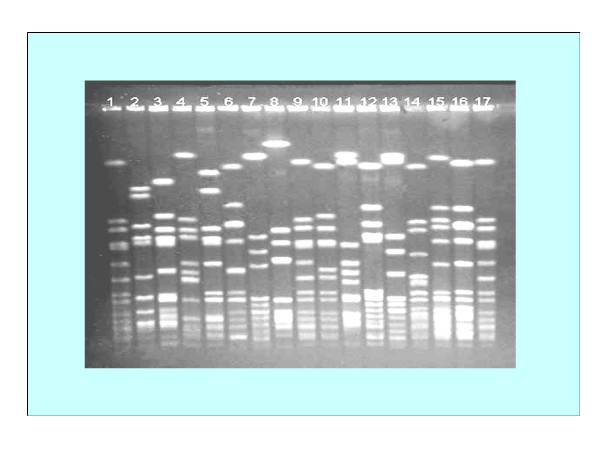
**Pulsed-field gel electrophoresis (PFGE) profiles of MRSA**. Fourteen MRSA fingerprint patterns were identified for the 61 strains of MRSA isolated in an Orthopaedic Surgical Ward. These strains were collected from wound infections (n = 56); patient's nose (n = 50); and nose of the hospital workers attending wound infections (n = 50). Lanes 1, 9 and 17 represent *S. aureus *8325 patterns for comparison.  Lanes 2 to 16 barring 9 respectively represent PFGE banding patterns A to N.

The 14 common methicillin – resistant *S. aureus *(MRSA) patterns were identified among 61 stains of MRSA isolated in the Orthopedic Surgical Ward (figure 4). The patterns were designated by the letters A to N based on difference in banding patterns. The banding type A was shown by highest number of MRSA strains (n = 7), where as type I was shown by only one MRSA isolate (Table 3). These results indicated that typing can be performed effectively through molecular techniques such as PFGE patterns but not through antibiograms as 61 strains showed 42 antibiograms that were narrowed down to only 14 types by PFGE. Moreover, it is evident that the MRSA strains which display a common antibiogram can not necessarily show the same PFGE pattern, for example, JMR-3, 55, 33, 59 showed antibiogram 3 (Table 3) but JMR-33 showed PFGE pattern L and JMR-59 showed PFGE pattern H although both JMR-3 and JMR-55 showed the same PFGE pattern A.

**Table 4:** Minimal inhibitory concentrations of A, G, Ak for *S. aureus *strain JMR10 and one *E. coli *transformant

**Strain**	**MIC (μg/ml)**		
	
	**A**	**G**	**Ak**
*S. aureus *strain JMR10	800	1000	200
*E. coli*	1	1	3
*E. coli *transformants	200	100	400

**Figure 5:**
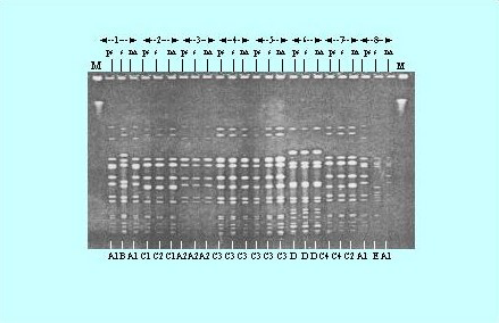
**Pulsed-field gel electrophoresis (PFGE) of MRSA clinical isolates**. PFGE patterns were indistinguishable between MRSA from the nasal cavity (na) and pus (ps) but the patterns were different for MRSA from the skin (s) and nearby wounds for cases 1 and 8. PFGE patterns were indistinguishable between MRSA from the nasal cavity and pus and closely related to that of MRSA from the skin for case 2. PFGE patterns were indistinguishable between MRSA from the skin and pus but closely related to that of MRSA from the nasal cavity for case 7. The patterns of MRSA isolates from the nasal cavity, skin, and pus were indistinguishable for cases 3, 4, 5, & 6.

Molecular typing by PFGE of *Sma*I-digested DNA from MRSA strains isolated from eight patients having postoperative wound infections admitted in Orthopedic Surgical Ward was performed (Fig 5). The staphylococcal strains were isolated from pus, skin and nose of the patients. The criteria proposed by Tenover et al [41] were employed to analyze the DNA fingerprints generated by PFGE. PFGE patterns are indistinguishable between MRSA from the nasal cavity (A1, A1), and pus (A1, A1) but the pattern is different for MRSA from the skin (B, E) nearby wounds for cases 1 and 8, respectively. PFGE patterns are indistinguishable between MRSA from the nasal cavity (C1) and Pus (C1) and closely related to that of MRSA from the skin (C2) for case 2 (Fig. 5). PFGE patterns are indistinguishable between MRSA from the skin (C4) and pus (C4) but closely related to that of MRSA from the nasal cavity (C2) for case 7. The patterns of MRSA isolates from the pus, skin and nasal cavity are indistinguishable for cases 3 (A2, A2, A2), 4 (C3, C3, C3), 5 (C3, C3, C3) and 6 (D, D, D). These results indicated that self-infection through colonization needs to be taken into consideration and the appropriate measures should be followed to minimize the role of carrier isolates in postoperative infections.

Furthermore the bacteria that normally colonize the human body (the resident microflora) could act as reservoirs for resistance genes, which could then be transferred to pathogens during their temporary colonization of the same site, and need to be focused while treating infections.

### Plasmid-determined resistance transfer

**Figure 6:**
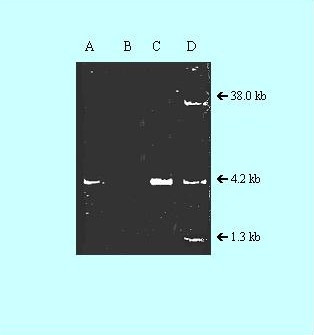
**Agarose gel electrophoresis of crude plasmid DNA**. Lane A, MRSA, resistant to A, G and Ak, having 4.3 kb plasmid (pJMR10); Lane B, *E. coli *(DH5α) sensitive to A, G, and Ak; Lane C, Transformant *E. coli *(DH5α) having 4.3 kb plasmid; Lane D, *S. aureus *RN834, containing the plasmid molecular markers.

The *E. coli *(DH5α), which was earlier sensitive to Ampicillin (A), Gentamicin (G) and Amikacin (Ak), now acquired resistance to these three antibiotics after transformation of plasmid pJMR-10 into it. Again plasmid was isolated from transformed *E. coli *(DH5α) and the isolated plasmid preparation from *S. aureus *(JMR-10) and transformed *E. coli *(DH5α) were loaded in 0.7% agarose gel. From Fig. 6 it appeared that the 4.3 kb plasmid from *S. aureus *(Lane A) was transformed to *E. coli *(Lane C). Minimum inhibitory concentrations of A, G, Ak on *S. aureus *strain JMR-10 and *E. coli *transformant indicated (Table 4) that the MIC for Ak was higher in *E. coli *than *S. aureus *and may be due to the fact that Ak resistance gene was very efficiently expressed in *E. coli*.

**Figure 7:**
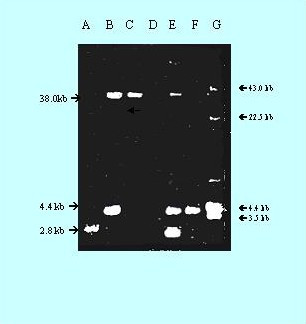
**Plasmids transferred in conjugation through mixed culture test (MCT) experiments (broth matings)**. Lane A, Carrier *S. aureus*, JN-49 (A^R^, P^R^, Cf^R^, E^R^, G^S^, T^S^), having 2.8 kb plasmid. Lane B, Clinical *S. aureus*, JS-105 (T^R^, E^R^, G^R^, A^S^, P^S^, Cf^S^), having 38 kb and 4.4 kb plasmid; Lane C, Cured trasnconjugant, JNS-1A (G^R^, E^R^, Cf^R^, T^S^, A^S^, P^S^), having 38 kb plasmid; Lane D, Cured trasnconjugant, JNS-1B (E^R^, Cf^R^, T^S^, G^S^, A^S^, P^S^), having no plasmid; Lane E, Transconjugant JNS-1 (T^R^, Cf^R^, E^R^, G^R^, A^R^, P^R^), having 38 kb, 4.4 kb and 2.8 kb plasmid; Lane F, Cured Transconjugant JNS-1C (T^R^, Cf^R^, E^R^, G^S^, A^S^, P^S^), having 4.4 kb plasmid; Lane G, Plasmid marker, *S. aureus *WBG 4483.

Plasmids were electrophoresed (Fig. 7) after isolation from JN-49 (Lane A), JS-105 (Lane B), and transconjugant JNS-1 (Lane E). Here transconjugants were screened as (A^R^, T^R^). The transconjugant JNS-1 was subjected to curing treatment with ethedium bromide. Three types of cured transconjugents were obtained. As shown in figure 7 cured transconjugant JNS-IA (Lane C), having 38 Kb plasmid showed resistance pattern as G^R^, E^R^, Cf^R^, T^S^, A^S^, P^S^. Similimarly cured transconjugant JNS-IB (Lane D), having no plasmid depicted resistance profile as E^R^, Cf^R^, T^S^, G^S^, A^S^, P^S^. Likewise, cured transconjugant JNS-IC (Lane F), having 4.4 Kb plasmid, displayed resistance pattern as T^R^, Cf^R^, E^R^, G^S^, A^S^, P^S^. The conjugation experiments clearly showed that resistant markers G and T got transferred from clinical *S. aureus *(JS-105) to carrier *S. aureus *(JN-49). Moreover the ciprofloxacin (Cf) and erythromycin (E) resistance seemed to be chromosomal mediated as evidenced in Lane D.

## Discussion

There are reports of emergence and high occurrence of strains resistant to methicillin from various parts of the world [29]. Recent studies have documented the increased costs associated with MRSA infection, as well as the importance of colonization pressure [42,43]. Already multiresistant to different classes of antibiotics, MRSA had been reported to acquire resistance to gentamicin and related aminoglycosides [30], therefore the treatment of infections due to these organisms and their eradication is very difficult. Constant monitoring of these strains is essential in order to control their spread in the hospital environment and transmission to the community. Of the 513 clinical strains of *S. aureus*, 180 (65.1%) were methicillin-resistant *S. aureus *(MRSA) and out of 337 coagulase-negative staphylococcal isolates 76 (22.5%) were methicillin-resistant (MRCNS). Of the 180 MRSA strains the highest number was found in pus followed by urine and blood. Of the 76 MRCNS studied the highest number was obtained from urine followed by conjunctiva and nasal swabs of patients having ocular infections. The number of MRSA isolates being drastically high in wound infections, this might be due to the fact that orthopedic unit is a fertile environment for MRSA. The open wounds and the frequent dressing changes often necessitate a dressing team or multiple persons plus the inherent immunosupression of the wound patients might lead to MRSA colonization.

The present study suggests that MRSA most likely remains a hospital-acquired infection, but a significant proportion may be acquired in community facilities like nursing and residential homes [43]. The major reservoir of staphylococci in hospitals are colonized/infected in-patients and colonized hospital workers, with carriers at risk for developing endogenous infection or transmitting infection to health care workers and patients [2,3,17-19,44], while transient hand carriage of the organism on the hands of health care workers account for the major mechanism for patient to patient transmission [20]. Low prevalence of MRSA colonization in an adult outpatient population indicated that MRSA carriers most likely acquired the organism through contact with healthcare facilities rather than in the community [45]. These data show that care must be taken when attributing MRSA colonization to the community if detected in outpatients or during the first 24 to 48 hours of hospitalization. The risk to patients in terms of transmission of MRSA seems to be influenced strongly by the proportion of patients with colonization at intensive care unit admission and is associated with severity of illness, length of stay, and exposures to antibiotics and medical devices [46].

Colonization with *S. aureus *can occur soon after birth and at any given time, the nasal carriage rate in adults is estimated at between 20% and 40% [47]. Healthcare workers have a higher incidence of colonization. The carrier state is clinically important because carriers undergoing surgery will experience more infections than non-carriers. Fierobe et al [42] has shown a relationship between MRSA postoperative intra-abdominal sepsis and nasal colonization of MRSA. MRSA strains are usually introduced into an institution by an infected or colonized patient or by a colonized healthcare worker, and transfer from one patient to another has led to major epidemics in tertiary care hospitals as well as chronic care facilities [3]. Colonization of anterior nares with MRSA carries a significantly higher risk for infection than does colonization by sensitive strains [18,47].

It is imperative we continue with basic infection control principles like hand washing and contact isolation and barrier nursing. Intranasal 2% mupirocin applied twice daily for five days appears to be the most effective topical agent against MRSA [48-50] and eliminates 91% of stable carrier states. By confining the investigation to nasal carriage in healthy state, colonization at other body sites may have remained undetected and the 'true' prevalence in this study may have been underestimated. There is, however, overall agreement that sensitivity of nose swabs in detecting MRSA carriage is reasonably high (>70%) [51-53], and it was decided to confine the investigation to nasal specimens for reasons of accessibility, compliance and consistency with other investigations. The design of the study does not allow one to distinguish between patients who acquired MRSA during their current episode of hospital stay or patients already colonized on arrival to the hospital. Despite this, most of the carriers appeared to have acquired the organisms in hospital settings, as most of the isolates seem to be of the same types of strains as isolated from nose of the hospital workers.

From this study we suggest that all patients having history of previous hospital admission and patients admitted directly from nursing homes should be screened for MRSA prior to any elective surgical or orthopedic operative procedures. We believe that patients for routine elective procedures should have a negative result before undergoing the procedure. If surgery cannot be delayed due to medical reason then prophylaxis against MRSA should be given prior to the operation, though we appreciate this may not have much benefit in case of emergency admissions. However we would suggest that in patients with any of the above risk factors admitted for emergency surgery which may involve prosthetic implant, should be screened as well as given MRSA prophylaxis prior to their operations.

Broad-spectrum insusceptibility of all 180 MRSA and 76 MRCNS isolates to common antimicrobial agents were observed. Among all 17 antimicrobial agents used only vancomycin was shown to be consistently effective against all MRSA and MRCNS. We did not find any glycopeptide resistant *S. aureus *in our study, and vancomycin and teicoplanin remain the drugs of choice, although decreased susceptibility as well as resistance to vancomycin has been reported recently [54,55].

Of the 850 stephylococcal isolates, surprisingly about 90%, 80% and 50% strains were resistant to Pencillin G, ampicillin and co-trimoxazole, respectively. This might be due to the fact that at present time these agents are tremendously used in the treatment of general infections. On the contrary to the multiple resistance of MRSA, MSSA seemed to be much more susceptible to all tested antimicrobial agents except for pencillin, ampicillin and co-trimoxozable. Our observation was that resistance in carrier isolates was lesser than clinical strains in case of all antibiotics tested. In the present study it is documented that resistance to chloramphenicol and ciprofloxacin was comparatively higher in ocular isolates as compared to staphylococcal strains isolated from other clinical sources. This might be due to more pronounced use of these antibiotics for treatment of ocular infections. This study underscores the need for hospital clinicians to be aware of the common bacterial isolates in their unit and their usual antibiotic susceptibility. This is imperative in order to make rational decisions for the prudent use of antibiotics, particularly for empirical therapy. Another important cause of resistance is excessive or inappropriate use of antibiotics in hospitals [56]. The magnitude of the problem of multi-resistance is such that clinicians must be familiar with the causes of antibiotic resistance and the measures for preventing or minimizing the emergence of resistance.

PFGE, because of its great discriminatory power and high degree of specimen typeability is accepted as the gold standard for the molecular typing of *S. aureus *isolates. It has successfully been used to study the epidemiology of *S. aureus *nosocomial infection and methicillin resistance [40,57-59]. Nevertheless, PFGE is time-consuming and labor intensive, in this study PFGE exhibited superiority as a technique for analyzing epidemiology of *S. aureus*. In the present study it was observed that the 61 strains of MRSA isolated in an Orthopaedic Surgical Ward displayed 42 patterns of antibiogram. These 61 MRSA strains were subjected to PFGE analysis whereby 14 PFGE patterns were observed. These results suggest that most of the MRSA appeared to have been acquired by patients during their current episode of hospital stay. Moreover similarity of PFGE patterns of MRSA isolates from pus, skin and nasal cavity suggest that most MRSA types isolated from pus were derived from the nasal cavity but some types were derived from the nearby skin and that these microorganisms occasionally cause wound infections.

MRSA acquisition depends on 2 major and independent determinants: colonization pressure and antimicrobial selective pressure. In case of colonization with distinct multiple clones of MRSA, antimicrobial pressure plays a major role; in the case of colonization with a single dominant clone of MRSA, colonization pressure plays a major role. McGown [60] proposed a biological model to explain the relationship between antimocrobial use and the emergence of resistance. At the level of individual patient, antimicrobial treatment leads to a large modification in the endogenous flora. The usual result is that susceptible strains are replaced by resistant ones. At the collective level, antimicrobial use in a hospital unit tends to maintain the presence of multidrug – resistant organisms in inpatients, healthcare workers, and the environment. In cases in which basic infection-control practices are inconsistently applied, these pathogens are implicated in the majority of infections. Antimicrobials such as β-lactams and fluoroquinolones, which are ineffective against MRSA and have excellent tissue diffusion, could promote the acquisition of MRSA by increasing the 'receptiveness' of the patients and thereby allowing the progression towards colonization and infection.

The spread of resistance to antimicrobial agents in *S. aureus *is largely due to the acquisition of plasmids and/or transposons [61]. Although transfer of resistance between staphylococcal strains in the laboratory has been shown to occur via transformation, transduction, and conjugation [62] only conjugative transfer appears to be significant in vivo [63]. In staphylococci, the conjugative transfer of resistant determinants is usually mediated by conjugative plasmids [64] but has also been shown to occur in the absence of detectable conjugative plasmids [65]. Conjugative plasmids, usually 35 to 50 kb, spread resistance determinants between species and genera [66,67]. Besides transferring the resistance determinants, they can mobilize non-conjugative plasmids [64] recombine with nonconjugative plasmids to form new plasmids or acquire and transfer resistance transposons [61,63]. Studies with human staphylococcal strains indicate that *Staphylococcus epidermidis *is a reservoir of antibiotic resistance genes that can be transferred to *S. aureus *under *in vitro *and *in vivo *conditions [64]. *In vitro *studies of drug resistance transfer between clinical and carrier staphylococcal strains, was done in the present study. The results indicated that tetracycline resistance was transferred from clinical to carrier isolates. This reflects that the use of antibiotics in humans to treat infections can promote resistance in normal flora.

The widespread occurrence and dissemination of resistance markers leading to multiple antibiotics ineffective, thus increasing the cost of health care, needs to be tackled logistically by wise and judicious use of existing antibiotics and by developing ideal and cost effective antibiotics having least chances of acquiring resistance. Furthermore the hospital acquired MRSA infections through colonization of patients and hospital workers demands appropriate and timely measure to counteract this health problem.

## Competing interests

The author(s) declare that they have no competing interests.

## Authors' contributions

JAD carried out all the microbiological, molecular and clinical laboratory experiments as a part of his PhD thesis and has written the draft manuscript. MAT and JAK provided strains and helped in the design of the study. AA and MAK coordinated and participated in design of the study. MR and KHB participated in the design and coordination and helped in the draft of the manuscript. MJD and NA helped in design, data analysis and interpretation and planning of the molecular part of the study. SA provided overall leadership, laboratory infrastructure and coordination, and corrected the manuscript.

All the authors read and approved the manuscript.
